# Efficacy and safety of pancreatic enzyme replacement therapy on exocrine pancreatic insufficiency: a meta-analysis

**DOI:** 10.18632/oncotarget.21659

**Published:** 2017-10-07

**Authors:** Can Gan, Yan-Hua Chen, Ling Liu, Jin-Hang Gao, Huan Tong, Cheng-Wei Tang, Rui Liu

**Affiliations:** ^1^ Division of Peptides Related with Human Diseases, State Key Laboratory of Biotherapy, West China Hospital, Sichuan University, Chengdu, China; ^2^ Department of Gastroenterology, West China Hospital, Sichuan University, Chengdu, China

**Keywords:** pancreatic enzyme replacement therapy, exocrine pancreatic insufficiency, efficacy, safety, standardization

## Abstract

**Background:**

Pancreatic enzyme replacement therapy (PERT) is widely applied to patients with exocrine pancreatic insufficiency (EPI), but its effect and safety has not been quantified. Therefore we performed a meta-analysis to determine the efficacy and tolerance of PERT on patients with EPI.

**Materials and Methods:**

PubMed, Medline, Cochrane library database, Evidence-based medicine/clinical trials published before December 2016 were searched by two independent reviewers to identify prospective randomized controlled trials (RCTs).

**Results:**

Seven RCTs, randomizing a total of 282 patients, were filtrated and assessed qualitatively (Jadad score). PERT increased CFA (WMD: 26.56, 20.35 to 32.76, I^2^ **=** 79.6%, *P* < 0.001) compared with baseline, and CFA (WMD: 17.97, 12.61 to 23.34, I^2^ = 76.7%, *P* < 0.001) vs. placebo. Meanwhile, CNA, SFE, SNE and SW were significantly improved in PERT compared with baseline and placebo, with no statistical differences in adverse events. Subgroup analysis indicated that standard forms of PERT displayed more effectiveness with significantly decreased heterogeneity, and large sample size also reduced the heterogeneity to some degree.

**Conclusions:**

PERT is demonstrated to be effective and tolerable in patients with EPI, especially using standard administration of PERT. Larger and higher quality studies on EPI are demanded to long-term effect of standard PERT treatment.

## INTRODUCTION

Exocrine pancreatic insufficiency (EPI), characterized as decreased synthesis or secretion of pancreatic enzymes and bicarbonate, occurs with parenchyma dysfunction or reduction or the ductal obstruction due to preexisting pancreatic diseases, leading to the maldigestion of food and subsequently malabsorption of nutrients [1, 2]. The common clinical symptoms remain recurrent abdominal pain, flatulence and weight loss, accompanied with typical steatorrhea in the case of pancreas lipase output decrease to < 10% of normal [3]. The diagnosis of EPI can be made on the basis of clinical manifestation besides auxiliary examinations: direct and indirect pancreatic function tests. The former containing endoscopic function testing, secretin-magnetic resonance pancreatography and secretin-endoscopic ultrasonography is sensitive but expensive and inaccessibility, the latter testing the contents of undigested food and stool is widely used for its convenience and facility [4–6].

Moreover, continuous malabsorption has a long-term negative effect on multiple systems, for example nyctalopia, cerebellar ataxia, increased prothrombin time and osteoporosis, increasing morbidity and mortality related to malnutrition [7, 8]. While the main causes of EPI remain chronic pancreatitis (CP), cystic fibrosis (CF) and pancreatic surgery (PS) [9].

Chronic pancreatitis, a chronic progressive inflammatory syndrome with irreversible damage on pancreatic parenchyma, mainly results from alcohol exposure, smoking, hyperlipidemia, genetic mutation and autoimmune diseases, [10] often manifesting the clinical signs of EPI. Cystic fibrosis, common life-shortening genetic disease in white individuals, affects epithelial secretory tissues and results in pancreatic and pulmonary dysfunction, with symptoms of EPI, such as poor weight, growth restriction and gastrointestinal symptoms [11]. Pancreatic surgery including partial and total resection, is usually used in the administration of pancreatic neoplasm or chronic pancreatitis. But it is still unclear whether postoperative pancreatic dysfunction derives from the extent of resection or the underlying pancreatic disease or the combination of both [1].

Pancreatic enzyme replacement treatment (PERT), a mixture of the digestive enzymes amylase, lipase, and protease, are widely utilized in management of EPI as an exogenous supplement whatever etiology for its efficacy, safety and toleration. PERT contains enteric-coated and non-enteric-coated capsules minimicrospheres, microspheres and tablets with various doses. As oral administration of pancreatic enzymes is vulnerable to gastric juice, enteric-coated capsules can protect the contents when transit through the stomach without acid-degradation to effectively release in duodenum with chyme. Minimicrospheres and microspheres, each with only a few millimeters in diameter, disperse more evenly and rapidly in chyme compared with conventional tablets [12].

However, therapeutic effects of PERT still remain equivocal. Yaghoobi M [13] conducted a systematic review that PERT failure to relieve abdominal pain in patients with CP. Whereas PERT were testified to be safe and effective in a systematic review and meta-analysis [14]. Another systematic reviews concluded that PERT cannot normalize the fat malabsorption, [15, 16] or the impacts on CP or CF are incomplete and inconsistent [17, 18]. Hence, to better understand the effect of PERT for EPI, we synthesized published RCTs via a meta-analysis, to quantitatively evaluate the coefficient of fat absorption (CFA), coefficient of nitrogen absorption (CNA), stool fat excretion (SFE), stool nitrogen excretion (SNE), stool weight (SW), abdominal pain and adverse events by comparing PERT with placebo.

## RESULTS

**Figure 1:**
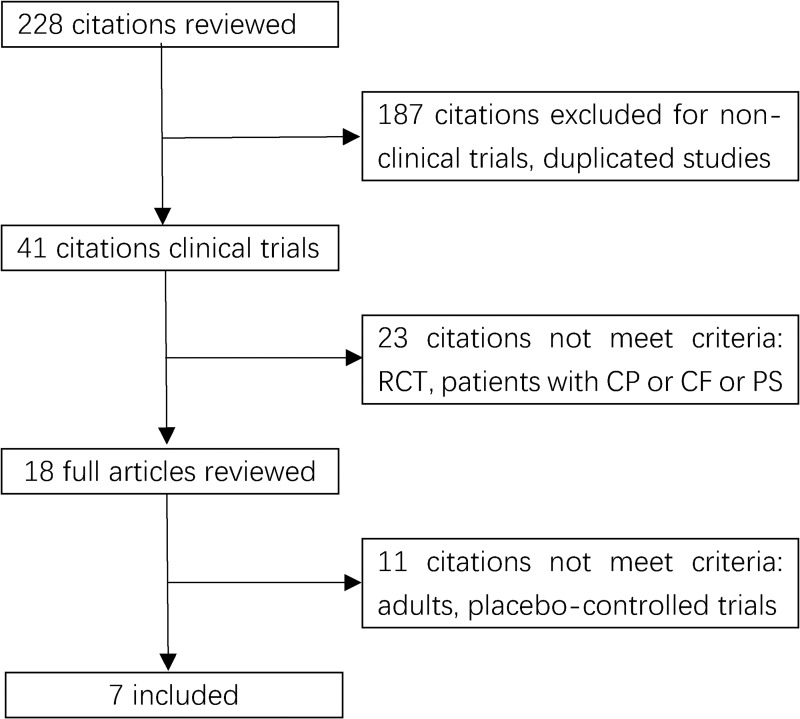
Flow chart of study selection RCT: randomized controlled trials. CP: chronic pancreatitis. PS: pancreatic surgery

A total of 228 studies were identified according to the searching strategy about pancreatic enzyme replacement therapy and exocrine pancreatic insufficiency. And seven RCTs met the criteria, with 282 patients (149 in the PERT group and 133 in the placebo group) randomized. Figure 1 depicts the PRISMA flow diagram.

### Characteristics and quality of included studies

The basic characteristics of included studies are shown in Table 1. Of the seven RCTs, four were from multicenter [19–22] and the rest from single, [23–25] while six were parallel design [19–22, 24, 25] and one cross-over design [23]. Three studies were in Europe, [22–24] one in the USA, [19] one in India, [21] one in South Africa [25] and one in the USA and Europe [20]. The definition of EPI caused by chronic pancreatitis or pancreatic surgery are presented: CFA < 80%, [21, 22] CFA < 80% or SFE > 10 g/d, [19] SFE > 15 g/d, [20] SFE ≥ 10 g/d, [24, 25] SFE > 7 g/d [23]. Five studies included post pancreatic surgery patients [20–22, 24, 25]. And quality of each study was assessed by Jadad score in Table 2 (six studies high quality, [19–24] one low quality [25]).

**Table 1:** Basic characteristics of included studies

Author, Year	Country	Duration	Study design	Inclusion criteria	Etiology	No of post-ps	Age	Patients randomized (male/female)
Halgreen, 1986	Denmark	NR	Single, randomized, double-blind, placebo-controlled, cross-over	Adults, CP, reduced EPF, with a meal-stimulated duodenal lipase concentration of less than 50 kU/L and a SFE > 7 g/day.	A4; I3 O3; H1	NR	29-59 (51)	11(6/5)
Paris, 1993	France	June 1986 to June 1987	Single, randomized, double-blind, placebo-controlled, parallel	Radiologically or perioperatively confirmed CAP, Previous steatorrhea ≥ 10 g/24 h).	A 60	R, not clearly	47	41(35/6)
O’Keefe, 2001	South Africa	NR	Single, randomized, placebo-controlled, parallel	≥ 18 y, evidence of CP (CT, US, ERCP et al) and EPI(SFE > 10 g/day in run-in phase)	A 27; I 2	9	49.1 ± 1.8 years vs. 57.8 ± 2.1	29(28/1)
Safdi, 2006	USA	NR	Multicenter, randomized, double-blind, placebo-controlled parallel	≥ 18 y, documented CP, EPI(CFA < 80% and/or SFE > 10 g/day in run-in phase)	NR	NR	Placebo 51 PERT 51.9	27(9/18)
Whitcomb, 2010	USA& Europe	April 2007 to August 2008	Multicenter, randomized, double-blind, placebo-controlled parallel	≥ 18y, EPI (stool elastase < 100 μ g / g, or SFE > 15 g/day in run-in phase), CP(radiographically or histologically proven) total or partial pancreatectomy > 180 days	NR	14	PERT 52 Placebo 50.6	54(39/15)
Thorat, 2012	India	June 2008 to May 2010	Multicenter, randomized, double-blind, placebo-controlled parallel	≥ 18y, EPI(CFA < 80% during the run-in phase), CP(radiographically or histologically proven)	NR	18	PERT 42.6 Placebo 43.2	62(47/15)
Seiler, 2013	Europe	NR	Multicenter, Randomized, double-blind, placebo-controlled parallel	≥ 18y, severe EPI(baseline CFA < 80%)due to partial or total PR ≥ 6 months prior to study start, stable condition after surgery(Karnofsky index ≥ 70)	Pancreatic surgery	58	PERT 57.6 Placebo 59.3	58

CP: chronic pancreatitis. CAP: chronic alcoholic pancreatitis PS: pancreatic surgery. A: alcoholic. I: idiopathic. O: obstructive. H: hyperlipidemic. EPI: exocrine pancreatic insufficiency. EPF: exocrine pancreatic function. R: reported. NR: not reported. CFA: coefficient of fat absorption. SFE: stool fat excretion. PERT: pancreatic enzyme replacement therapy.

**Table 2:** Jadad score of included studies

Study	Randomization	Double-blind	Withdrawals and dropouts	Allocation concealment	Jadad score
Halgreen, 1986	1	1	0	1	3
Paris, 1993	1	1	1	0	3
O’Keefe, 2001	1	0	1	0	2
Safdi, 2006	1	1	1	0	3
Whitcomb, 2010	1	2	1	1	5
Thorat, 2012	1	2	1	1	5
Seiler, 2013	1	2	1	1	5

Patients enrolled first had to finish a run-in phase or wash-out phase to filter eligible participants before double-blind treatment phase. And stool collection was performed to detect the fat and nitrogen contents and stool weight. Intervention was given with placebo or PERT randomly, which contained main four types in enteric-coated microtablet or microspheres or minimicrospheres: Creon^®^ in four studies, [19–22] Pancrease^®^ in one, [23] Panzytrat^®^ [24] in one and a non-informed type in one study. [25] Creon^®^ and Pancrease^*®*^ have been approved by the US Food and Drug Administration, acknowledged to be standard [26]. Daily fat intake was recorded during both periods for calculating the fat absorption. Mean change of CFA increased in PERT compared with placebo statistical significantly in five studies. Adverse events incidences measured in five studies were developed in both PERT and placebo group. (Table 3).

**Table 3:** Clinical trials on treatment of pancreatic exocrine insufficiency

Study	Run-in phase	No of PERT	No of placebo	Fat intake/day(g)	Intervention			Time of stool fat collection	Mean change of CFA (pert vs. placebo)	Adverse events
					Type	Dosage(oral)	Time (days)			
Halgreen, 1986	14 days	11	11	100	Pancreaze^®^ EC, MS (L: 4000 NFU A: 20,000 NFU, P: 25,000 NFU)	Meal:2 capsules, tid. snack:1 bid.	14	2-day equilibration followed by 3-day collection	NR	NR
Paris, 1993	7–9 days	32	28	≥ 100	Panzytrat^®^ 25 000 EC, MT (L: 25000, A: 22500, P: 1250) Ph. Eur	meal:2capsules tid.	7	4-day equilibration followed by 3-day collection	23.7 vs. 19.7	PERT:12.5% Placebo:10.7%
O’Keefe, 2001	7-day placebo followed by 7-day PERT	15	14	100	Pancreatic enzyme supplement, EC, MMS (L: 10,000 USP U A: 33,200 USP U, P: 37,500 USP U)	Meal: 4 capsules tid. snack:2 bid.	14	4-day equilibration followed by 3-day collection in placebo period, 11-day equilibration followed by 3-day collection in PERT period	26.8 vs. 0, *P* = 0.002	NR
Safdi, 2006	14- day placebo	13	14	≥ 100	Creon 10 EC, delayed-release MMS (L: 10000 USP U, P: 37500 USP U, A: 33200 USP U)	Meal:4 capsules tid. snack:2 capsules bid.	14	11-day equilibration followed by 3-day collection	36.7 vs. 12.1, *P* = 0.0185	PERT:35.7%, Placebo:23.1%
Whitcomb, 2010	5-day placebo	25	29	≥ 100	Creon (pancrelipase)12,000 MMS USP U	Meal:6 capsules tid. snack:3 capsules bid.	7	2-day equilibration followed by 3-day collection in run-in phase	32.1 vs. 8.8, *P* < 0.0001	PERT:20.0%, Placebo:20.7%
Thorat, 2012	7-day followed by 7-day PERT	34	28	≥ 100	Creon 40000 MMS Ph. Eur	Meal: 2 capsules tid. snack: 1 bid.	7	4-day equilibration followed by 3-day collection	18.5 vs. 4.1, *P* = 0.001	PERT:35.3%, Placebo: 25.0%
Seiler, 2013	7-day followed by 7-day PERT	32	26	Reported, not given	Creon 25000 MMS Ph. Eur	Meal: 3 capsules tid. Snack:2 bid.	7	4-day equilibration followed by 3-day collection	21.4 vs. -4.2, *P* < 0.001	PERT:37.5% Placebo:26.9%

PERT: pancreatic enzyme replacement therapy. EC: enteric-coated MS: microspheres. MT: microtablet. L: lipase. A: amylase. P: protease. MMS: minimicrospheres. FA: fat absorption. SFE: Stool fat excretion. CFA: coefficient of fat absorption. NR: not reported. NFU: national formulary units. IU: international unit. USP U: United States Pharmacopeia units. Ph. Eur: European pharmacopoeia.

(1 USP = 1 Ph. Eur. = 1 NFU)

### Meta-analysis of main outcomes

**Figure 2:**
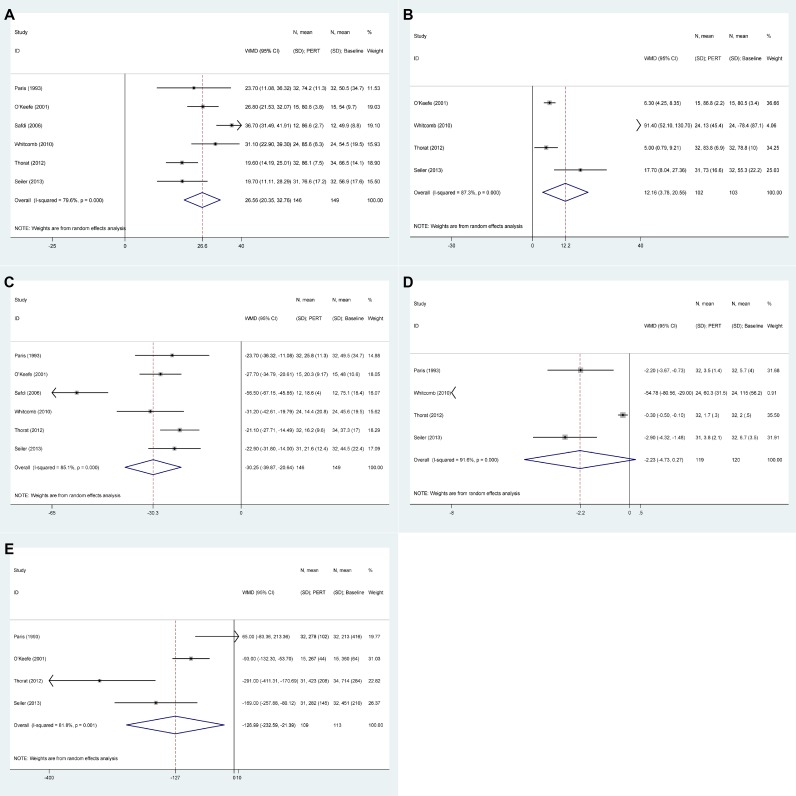
The pooled results of pancreatic enzyme replacement therapy versus baseline (**A**) Coefficient of fat absorption (CFA). (**B**) Coefficient of nitrogen absorption (CNA). (**C**): Stool fat excretion (SFE). (**D**): Stool nitrogen excretion (SNE). (**E**): Stool weight (SW).

Figure 2 shows the results of PERT group versus baseline. Overall results revealed that CFA increased in PERT versus baseline (WMD: 26.56, 20.35 to 32.76, *P* < 0.001) despite considerable heterogeneity (I^2^ = 79.6%), enhanced CNA in PERT versus baseline in four studies (*P* = 0.004), decreased SFE in six studies (*P* < 0.001), decreased SNE in four studies (*P* = 0.080), reduced SW in four studies (*P* = 0.018).

**Figure 3:**
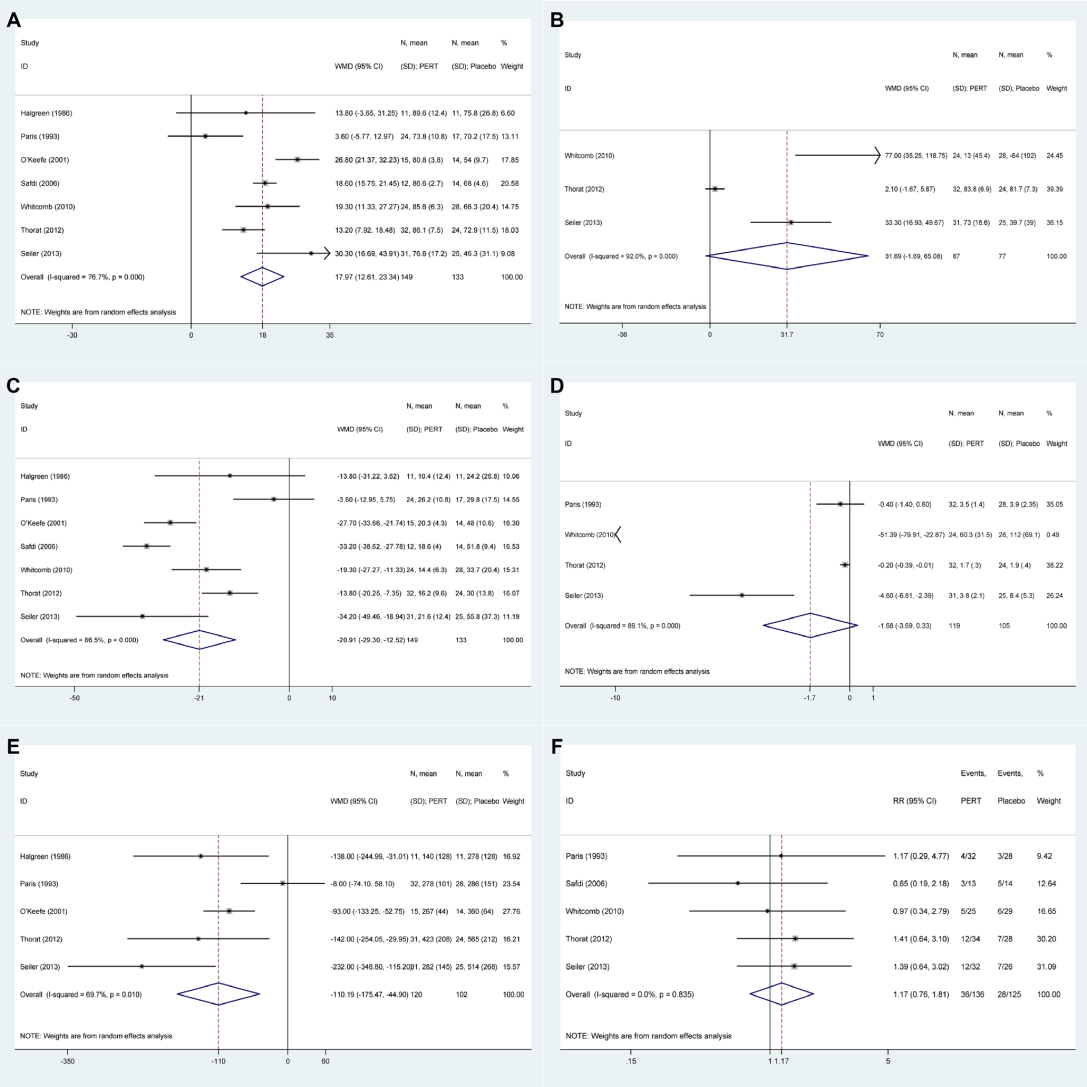
The pooled results of pancreatic enzyme replacement therapy versus placebo (**A**) Coefficient of fat absorption (CFA). (**B**) Coefficient of nitrogen absorption (CNA). (**C**) Stool fat excretion (SFE). (**D**) Stool nitrogen excretion (SNE). (**E**) Stool weight (SW). (**F**) Adverse events (AE).

Figure 3 presents the results of PERT versus placebo group. The pooled results declared enhanced CFA in PERT vs. placebo (WMD: 17.97, 12.61 to 23.34, *P* < 0.001) in spite of obvious heterogeneity (I^2^ = 76.7%), increased CNA in three studies (*P* = 0.063), decreased SFE in seven studies (*P* < 0.001), depressed SNE in four studies (*P* = 0.102), depressed SW in five studies(*P* = 0.001). Treatment-emergent adverse events manifesting abdominal pain/discomfort and flatulence during the treatment phase among PERT and placebo group did not reach significant differences (RR: 1.17, 0.76 to 1.81, *P* = 0.466) with I^2^ = 0.0% in five studies.

### Subgroup analyses

Due to high heterogeneity, we performed random effect analysis and subgroup analysis to explore the potential causes. (Table 4).

**Table 4:** Subgroup analysis of the form of PERT and the sample size

	Method	Subgroup	Study	WMD (95% CI)	Z	*P*	Heterogeneity		
							*P*	I^2^	Tau^2^
CFA	Form	Standardization	5	17.86 (13.83, 21.89)	8.68	0.000	0.148	41.1%	7.880
		Non-standardization	2	15.53 (–7.20, 38.25)	1.34	0.181	0.000	94.3%	253.850
	Sample	< 50	4	16.80 (8.42, 25.19)	3.93	0.000	0.000	83.9%	53.870
		> 50	3	19.06 (10.75, 27.37)	4.50	0.000	0.052	66.2%	34.501

#### Standard PERT forms versus non-standard PERT forms

Five studies using standard forms of PERT as a group was compared with non-standard group. CFA was improved more in standard forms with a statistically significant decreased heterogeneity (I^2^ : from 76.7% to 41.1%, *P* = 0.15).

#### Small sample (≤ 50) versus large sample (> 50)

Three studies were allotted to large numbers of patients, and four assigned to small. CFA was improved in large numbers with a decreased heterogeneity. (I^2^: from 76.7% to 66.2%, *P* = 0.05).

### Sensitivity analyses

#### Each trial’s contribution to the combined effect

**Figure 4:**
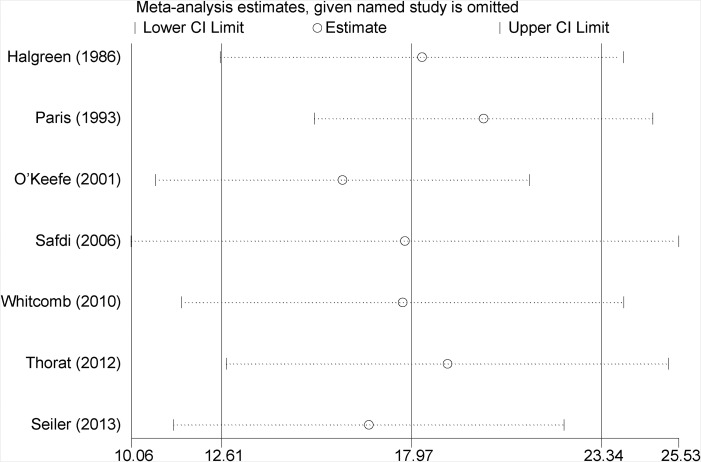
Leave-one -out analysis of pooled results of CFA between PERT and placebo group Meta-analysis random-effects estimates were used. The two ends of the dotted lines represented the 95% CI.

The remained therapeutic effect was still significant with high heterogeneity after excluding a study in turn. (Figure 4) Effect of the methodological quality of studies. The effect remained unchanged and robust with decreased heterogeneity after excluding a low-quality study (I^2^: from 76.7% to 67.0%, *P* = 0.01).

## DISCUSSION

In the meta-analysis we combined primary and secondary evidences from seven RCTs involving 282 patients with chronic pancreatitis or post pancreatic surgery, to evaluate the efficacy and safety of PERT for EPI. And we made several key observations.

First, PERT effectively improved CFA, CNA, SFE, SNE and SW in contrast to baseline and placebo. Second, adverse events between PERT and placebo group had no significant differences. These events mainly manifest mild to moderate gastrointestinal syndromes such as abdominal pain and flatulence, which may also associate with the underlying pancreatic diseases. In addition, standard forms of PERT and sample size contributed to the heterogeneity detected by subgroup analysis. Studies of standard PERT treatment performed higher beneficial effects on EPI without safety concerns with significantly decreased heterogeneity, which provided more stable and persuasive evidence. After grouping large sample sizes, the heterogeneity decreased though not significantly, it proved more steady and convincing results, which may enlighten larger size RCTs on the therapeutic effect of PERT.

Besides above, the pathogeny of exocrine pancreatic insufficiency might contribute to the heterogeneity. Various types and severities of diseases response and tolerate differently to the interventions. Alcoholic, idiopathic, obstructive or hyperlipidemic sources might form the basis of chronic pancreatitis. Pancreatic surgery may be involved in benign or malignant factors, and the type or extension of surgery. Each original RCT included CP or PS patients with diverse etiologies, but did not stratify or observe the outcomes based on the etiology, which may be relevant to the heterogeneity and bias.

It is a pity that RCTs of cystic fibrosis patients, including adolescents, were excluded owing to our inclusion criteria of adult patients. Creon or pancrelipase has been indicated for effective and safe treatment of steatorrhea in adult and adolescent patients with exocrine pancreatic dysfunction due to cystic fibrosis [27, 28]. And a novel purified PERT, liprotamase has been demonstrated to enhance fat and nitrogen absorption without safety concerns in CF patients [29]. Though the effects of PERT are testified by some studies, more extensive and comprehensive original researches about PERT are required to develop on CF to ensure consistent quality, efficacy and safety.

All RCTs described the run-in or wash out phase before the double mind treatment period, in which 72 h stool collection and analysis were performed to ensure eligible steatorrhea patients. After the phase, candidates had the same baseline. In addition, all the RCTs recorded the 72 h stool collection in run-in and randomized phase and daily fat intake. However, original studies merely conducted around seven days’ PERT before analyzing the outcomes without long-term treatment and follow-up to fat absorption and weight gain, though two studies developed 1-year open-label extensions and obtained increased body weight and BMI [22, 30]. The coefficient of fat absorption, used as the primary results though, in fact is inconvenient for an indirect clinical sign. The recommendation for the enteral nutrition after PERT of the European guidelines indicates the markers of treatment success as the weight gain or improvement of the steatorrhea [31]. Actually, weight gain need a long-time follow up and is a blunt way to assess the subtle and sensitive sign of nutrition [32]. Therefore, more high- evidenced RCTs about long term efficacy and safety of PERT are required.

Regrettably all RCTs included failed to attach importance to pancreatic diabetes, a complication of EPI. Pancreatic diabetes, also named type 3c diabetes, is distinguished from type 1 and 2 diabetes and causes endocrine dysfunction [33]. Pancreatic diabetes accounts for 1–2% of all types of diabetes in North America, [34] 0.8% in a Japan nationwide study, [35] and 15–20% in India and Southeast Asia for endemic fibrocalcific or tropical pancreatitis [36–38], in fact has higher incidence than generally known due to underdiagnosis and misdiagnosis. Pancreatic diabetes usually manifests as “brittle diabetes” with poor glucose control due to impaired insulin, glucagon and pancreatic polypeptide. The symptoms of hyperglycemia and hypoglycemia are common. Unfortunately, there is no high evidence RCTs or recommendations for the treatment of type 3c diabetes, hence whose treatment refers to type 2 diabetes. The injection of insulin for hyperglycemia should be cautious in case of hypoglycemia on account of decreased hepatic insulin sensitivity, enhanced peripheral insulin sensitivity and subsequently decreased secretion of glucagon [39]. However, the study showed hypoglycemia is particularly tough to manage. PERT may exacerbate glucose mechanism for the effect of increasing glucose absorption, but on the other hand, decrease the incidence of hypoglycemia [1]. Chronic pancreatitis and diabetes are regarded as the risk factors of pancreatic malignancy. Metformin, due to low incidence of hypoglycemia and anti-neoplasm effects, is hence recommended as the first line therapy for pancreatic diabetes [40, 41].

The use of oral exogenous pancreatic enzyme was not standardized, only Creon^®^ and Pancrease^®^ in the included studies are approved by FDA.26 There exists a large possibility that different types of PERT possess various properties and potencies: lipase concentration, formulation, oral time and so on. Lipase contents maintain the primary therapeutic effect of PERT. A variety of lipase doses, within the recommended dose ranges from reviews, are applied by different studies according to the preexisting diseases due to the lack of formalized PERT dose guidelines. High dose may improve more steatorrhea and abdominal pain in cystic fibrosis patients with no different side effects [42]. Higher dose than usually used might obtain more fat and nitrogen absorption, and consequently improve nutritional parameters in more severe CP patients [43, 44]. However, high strength PERT is demonstrated unimproved evident effectiveness but high expense and more adverse events, such as fibrosing colonopathy, hyperuricemia or potential for viral transmission [45–50]. Therefore, Cystic Fibrosis Consensus Committee provides a guideline that the upper limit of daily dosage of PERT in patients with cystic fibrosis: 10,000 IU lipase per kg body weight [51]. Moreover microspheres, with several millimeters in diameter, have lower risks of pyloric retention and diverse more rapidly, meanwhile decrease more steatorrhea and abdominal pain compared with tablets [12, 52]. However, minimicrospheres are not superior to microspheres in treatment of EPI conversely higher cost, but on the other hand because of smaller size enhance patient compliance [53]. The time of oral PERT is administrated differently in studies, but is recommended to take in enzymes during meals at least after meals for optimal effect [54]. Whereas, original researches included failed to record the time of PERT intake. Due to impaired pancreatic bicarbonate secretion and relatively high gastric acid secretion, fat malabsorption in some studies still persists in patents with EPI during the treatment of enteric-coated enzyme microspheres alone. However, addition of proton pump inhibitors with optimal dose of PERT, fat digestion and residual steatorrhea can be significantly improved or normalized in CP and CF patients to enhance the efficacy of PERT [55, 56]. Meanwhile, in patients after pancreatic surgery, the prevalence of peptic ulcer is about 5%, thus optimized therapy should be the combination of PPI and PERT [57]. Delayed gastric emptying, an early complication of post operation, is up to 40% in patients after Whipple’s surgery, and usually treated with prokinetic drugs. Fibrates or Statins are used to lower hyperlipidemia [58].

Unfortunately, though diverse PERT may perform different therapeutic effects by grouping the forms of PERT in this meta-analysis, we are unable to stratify and observe PERT in depth for unknown property and potency of nonstandard treatments, we also cannot use meta regression analysis to further explore the origin of heterogeneity owing to small numbers of RCTs. However, the strength of the analysis includes complete assessments of efficacy and safety of PERT at the same time the methodological quality of original studies. Though a meta-analysis by de la Iglesia-Garcia D also concludes the efficacy of PERT, fails to consider the influence of standard PERT on EPI [14]. However, in our meta-analysis, we find out standard treatment with more improved physical parameters, thereby consider standard administration of PERT as a factor associated with the therapeutic effect, which may support a novel insight for treatment meanwhile as a basis for the exogeneous pancreatic enzymes selection.

In summary, our meta-analysis indicates that pancreatic enzyme replacement therapy has beneficial effects and tolerance in patients with exocrine pancreatic insufficiency, and standard forms of PERT may be more effective, by quantitatively observing seven RCTs. Although the high risk of bias cannot be completely found out and ruled out, to some degree the outcomes may be regarded as a basis of the current evidence for patients and physicians. Above all, more pancreatic enzyme replacement therapies are required to be normalized, and more large size and high quality comprehensive researches should be conducted to better evaluate the long-term effect of pancreatic enzyme replacement therapy on exocrine pancreatic insufficiency.

## MATERIALS AND METHODS

### Search strategy

A literature search of related studies was conducted in the databases of Pubmed, Medline, Cochrane library, Evidence-based medicine/clinical trials published before December 2016, using the following key words: pancreatic enzyme replacement therapy, pancreatic exocrine supplement, pancreatic lipase, Pancreatin, Ultrase, Cotazym, Creon, rotilase, amylase, exocrine pancreatic insufficiency/dysfunction, chronic pancreatitis, cystic fibrosis, pancreatic resection/surgery, pancreatic cancer/tumor/neoplasm, alcohol, randomized controlled trials, placebo-controlled and RCTs. To ensure all relevant citations were included in this study, the reference lists from relevant articles were manually screened. The studies were limited to clinical trials and those written in the English language.

### Selection criteria

To be included in this meta-analysis, studies had to meet the following criteria: (1) study: prospective, randomized, placebo-controlled studies; (2) patients: ≥ 18 years old with confirmed EPI whatever etiology (chronic pancreatitis, cystic fibrosis, total or partial pancreatic resection et al); (3) intervention: oral PERT and placebo; (4) outcomes: the primary outcome: CFA, the secondary outcomes: CNA, SFE, SNE, SW, AE. Animal studies, abstracts, case reports, letters, expert opinions, editorials, reviews, meta-analyses, non-RCTs and duplicate studies were excluded.

### Data extraction and quality assessment

Relevant data, including the first author’s name, publication year, study design, quality score, number of patients, diagnosed criteria of EPI and CP, number of post pancreatic surgery or cystic fibrosis, the dosage and type of pancreatic enzyme, outcomes and adverse events were extracted by two reviewers. And each included study was assessed according to The Jadad score [59]. The criteria of this score was as follows: (1) randomization (yes = 1 point, no = 0); (2) double-blind (described = 2 points, mentioned but not described = 1 point, no = 0); (3) withdrawals and dropouts (yes = 1 point, no = 0); (4) allocation concealment (described = 1 point, no = 0). The quality score ranges from 0 to 5 points; a low-quality report score is ≤ 2 and a high-quality report score is at least 3.

### Outcomes assessed

The primary outcome was the coefficient of fat absorption (CFA), using fat intake (100 g/day) and excretion (72 hours stool collection) to calculate out according to the following equation: CFA (100%) = [(fat intake in g-fat excretion in g)/fat intake in g] ×100. The secondary outcomes contain CNA, stool fat excretion (SFE), stool nitrogen excretion (SNE), and stool weight (SW), stool consistency (formed/normal or soft/watery), stool frequency (stools per day), flatulence(none/mild/moderate/severe), abdominal pain (none/mild/moderate/severe) and adverse events. The CNA was calculated according to the following equation: CNA (100%) = [(nitrogen intake in g-nitrogen excretion in g)/nitrogen intake in g] ×100.

### Statistical analysis

Means and SDs of continuous variable were extracted from the forest plots using Stata MP 13 software (Cochrane Collaboration, Oxford, UK). Continuous variables were expressed as weighted mean differences (WMD) and discontinuous variables as RRs with 95% CIs. Statistical heterogeneity is calculated by I^2^ statistic, with value over 50% indicating substantial heterogeneity and *P* < 0.1 meaning significantly [60]. The random-effects model was adopted for the heterogeneity. Subgroup analyses stratified by potential confounding factors was carried out to explore the sources of heterogeneity. In addition, sensitivity analysis was performed by removing one study each time and examining the influence of a specific study on the pooled results [61].

## Abbreviations

PERT: pancreatic, Enzyme, replacement therapy
EPI: exocrine, pancreatic, insufficiency
RCTs: randomized controlled trials
CP: chronic pancreatitis
CF: cystic fibrosis
PS: pancreatic surgery
CFA: coefficient of fat absorption
CNA: coefficient of nitrogen absorption
SFE: stool fat excretion
SNE: stool nitrogen excretion
SW: stool weight
AE: adverse events
SDs: standard deviations
WMD: weighted mean differences
RR: relative risk
95% CI: 95% Confidence Interval
